# Ribosome-binding protein 1: A multidimensional regulator of cancer progression and a novel target for precision therapy (Review)

**DOI:** 10.3892/ol.2025.15358

**Published:** 2025-10-23

**Authors:** Ho Huang, Jia Ouyang

**Affiliations:** 1 Peking University Health Science Center, Peking University, Beijing 100044, P.R. China; 2 Department of Neurosurgery, Peking University People's Hospital, Beijing 100044, P.R. China

**Keywords:** cancer, endoplasmic reticulum, molecular mechanism, ribosome-binding protein 1, signaling pathway, unfolded protein response

## Abstract

Ribosome-binding protein 1 (RRBP1), a core regulator of endoplasmic reticulum-ribosome interactions, serves key roles in the development and progression of various cancer types by coordinating protein synthesis and organelle dynamic interactions. RRBP1 regulates the unfolded protein response by stabilizing glucose-regulated protein 78 and it enhances cancer cell adaptation to endoplasmic reticulum stress and chemotherapy. The stability of RRBP1 is regulated by N6-methyladenosine modification by methyltransferase-like 3 and deubiquitination by ubiquitin-specific processing protease 35. Furthermore, RRBP1 drives cellular anti-apoptosis mechanisms by activating pro-survival pathways such as TGF-β1/SMAD, PI3K/AKT and Notch or binding cyclic RNAs. By contrast, aberrant activation of kinase function and deubiquitination pathways by *RRBP1* fusion genes [*RRBP1*-anaplastic lymphoma kinase, *RRBP1*-Raf1 proto-oncogene, serine/threonine kinase and *RRBP1*-ubiquitin specific peptidase 6] exacerbates malignant progression. Furthermore, the pleiotropic regulation of RRBP1 in neurodegeneration, cardiovascular homeostasis and bone metabolism highlights its environment-dependent functions. The present review identified the multidimensional regulatory network of RRBP1 in cancer and non-cancer systems to enhance the understanding of its molecular mechanism, demonstrated its broad regulatory value and potentially provided a key entry point to analyze the disease and develop precision therapies.

## 1. Introduction

Cancer is among the most notable challenges to global public health because of its high prevalence, molecular heterogeneity and tendency towards therapy resistance. According to the GLOBOCAN 2022 report, an estimated 19.96 million new cancer cases and 9.74 million cancer deaths occurred worldwide in 2022 (1). The identification of the following 14 cancer hallmarks in human tumors has markedly influenced the understanding of malignant progression in recent decades (2,3): Sustaining proliferative signaling, evading growth suppressors, resisting cell death, enabling replicative immortality, inducing or accessing vasculature, activating invasion and metastasis, deregulating cellular metabolism, avoiding immune destruction, genome instability and mutation, tumor-promoting inflammation, senescent cells, polymorphic microbiomes, nonmutational epigenetic reprogramming and unlocking phenotypic plasticity. Despite major advances in targeted therapies and immuno-oncology, clinical treatments continue to face three major obstacles: i) Intrinsic and acquired therapy resistance; ii) recurrence driven by the reactivation of dormant cancer cells; and iii) metastasis resulting from tumor evolution. These challenges highlight the urgent need for novel therapeutic targets and precision interventions to improve clinical outcomes in the future.

Ribosome-binding protein 1 (RRBP1), a key protein in endoplasmic reticulum (ER)-ribosome binding, serves pleiotropic roles in tumorigenesis and progression. Through its regulation of organelle dynamics and protein synthesis, RRBP1 promotes malignant phenotypes such as cell proliferation, invasion, metastasis and chemoresistance. Notably, RRBP1 is upregulated in various cancer types, such as breast cancer, colorectal cancer and bladder cancer, and its expression levels are positively associated with advanced clinical stages and poor prognosis (4–10). Understanding the molecular mechanisms of RRBP1 provides insights into dysregulated organelle communication and offers a potential foundation for the development of therapies targeting subcellular interactions. The present review systematically describes the roles of RRBP1 in cancer, emphasizing its functional diversity. It first outlines the structural features and functions of RRBP1, then analyzes its expression and regulatory mechanisms across several cancer types. Lastly, it evaluates the potential of RRBP1 as a therapeutic target and proposes combined strategies targeting organelle interaction networks to potentially enhance treatment efficacy, with the goal of expanding future research directions in precision oncology.

## 2. Function of RRBP1

**Figure 1.**
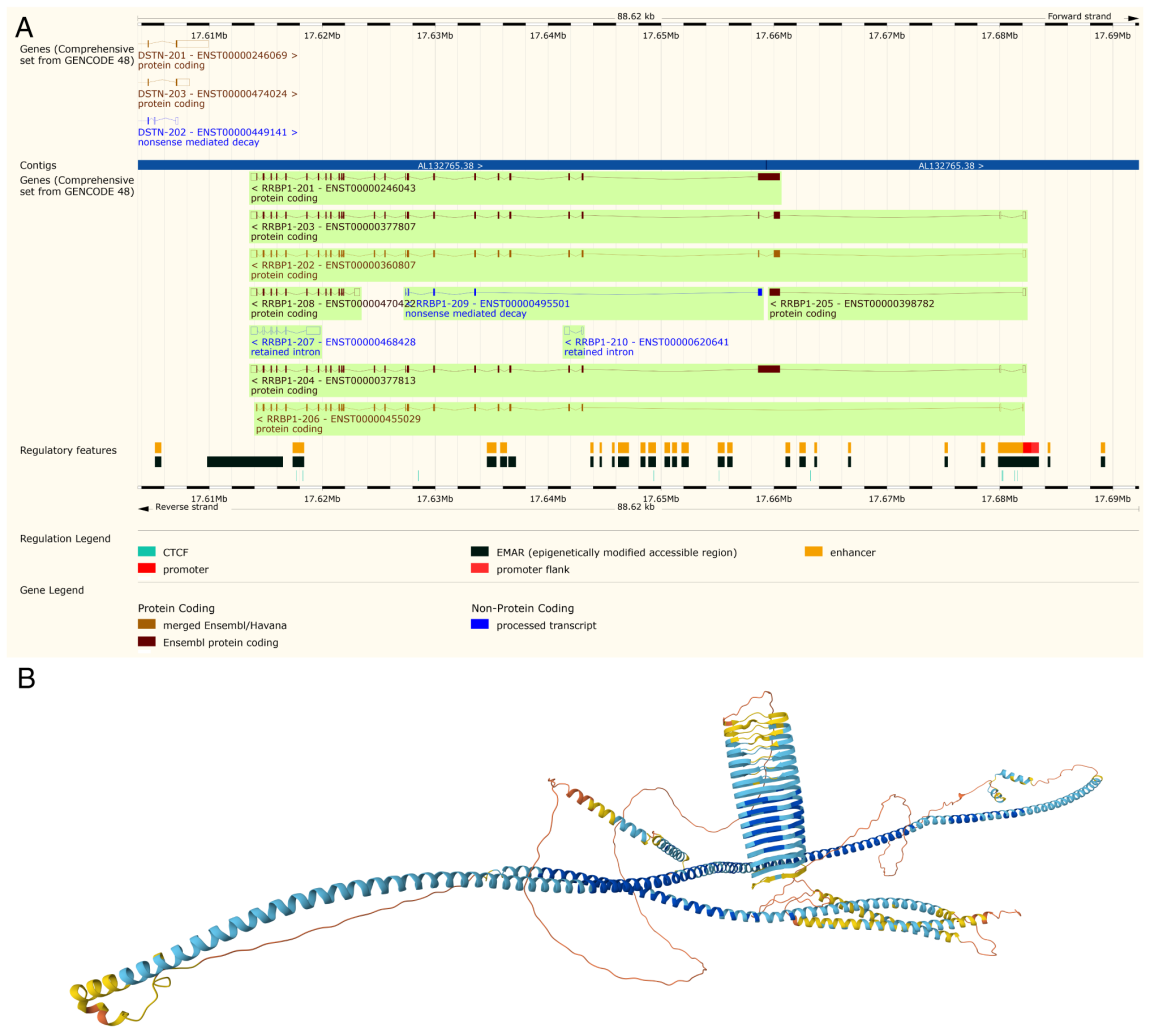
Schematic of *RRBP1* gene structure and predicted 3D protein conformation. (A) *RRBP1* gene from Ensembl, revealing various splicing variants, coding and non-coding transcripts and regulatory features. The default transcript (MANE Select) is RRBP1-204. Visualization adapted from the Ensembl Genome Browser (RRBP1, release 114, EMBL-EBI, http://www.ensembl.org). (B) Predicted protein structure of RRBP1. The color gradient from blue to yellow indicates the predicted local distance difference test confidence score (blue, high confidence; yellow/orange, low confidence). The structure reveals the characteristic long α-helical segments and coiled-coil architecture of RRBP1. Predicted protein structure from the AlphaFold Protein Structure Database (UniProt ID, Q9P2E9, DeepMind and EMBL-EBI, http://alphafold.ebi.ac.uk). RRBP1, ribosome-binding protein 1; EMBL-EBI, European Molecular Biology Laboratory-European Bioinformatics Institute; MANE, matched annotation.

RRBP1, also known as p180 because of its molecular weight, is a ribosome-binding protein that localizes in the ER membrane and is essential for the transport of newly synthesized proteins (11). The gene structure of RRBP1, featuring multiple splicing variants and its predicted protein architecture, is detailed in Fig. 1. The present review used the Ensembl Genome Browser (release 114; http://www.ensembl.org/) to display the genomic structure of RRBP1, highlighting its splice variants, including the MANE select transcript RRBP1-204 (Fig. 1A). Then the present review used AlphaFold (https://alphafold.ebi.ac.uk/) to predict the 3D protein structure of RRBP1 (UniProt ID, Q9P2E9), emphasizing its characteristic long α-helical segments and coiled-coil architecture (Fig. 1B). RRBP1 consists of a hydrophobic NH_2_-terminus and an acidic coiled COOH-terminal structural domain. The former includes a transmembrane structural domain and a highly conserved tandem repeat sequence (ribosome-binding structural domain), whereas the latter contains a lysine-rich region and a PDZ-binding structural domain (12–14). These structures enable RRBP1 to interact efficiently with ribosomes, ER and other cellular components.

### Role of RRBP1 in the regulation of protein synthesis and translation

Although RRBP1 was originally described as a ribosomal receptor, this type III transmembrane protein on the ER is also specifically involved in ER-dependent translational regulatory mechanisms, such as the mRNA localization of placental alkaline phosphatase, collagens Iα1 plus Iα2, IIIγ and calreticulin, by directing their binding to ER membranes (15). Furthermore, RRBP1 promotes the localization and anchoring of specific mRNAs through a ribosome-independent mechanism (16). RRBP1 regulates the assembly of multimer/protein complexes through its C-terminal structural domain, thereby promoting translational activity (17). In collagen secretory cells, RRBP1 preferentially enhances the efficiency of secretory protein biosynthesis by strengthening the association of ribosomes with the ER membrane and it promotes the efficiency of multimer formation, thus highlighting its central role in efficient translation in specialized secretory cells (17). Notably, the finding that RRBP1 deletion induces compensatory upregulation of signal recognition particle (SRP) receptor subunits (SRα/SRβ) potentially indicates a functional complementarity with SRP-dependent pathways (15). However, since the ER binding rate of Sec61β mRNA was not markedly altered after knockdown of RRBP1, its ER localization may be achieved through a pathway independent of RRBP1 and multiple mechanisms may coordinate the targeting and synthesis of tail-anchored proteins (18). For example, in the case of Sec61β mRNA, localization can occur independently of active translation yet is enhanced by translation, its ORF sequence appears to harbor elements mediating ER anchoring and some transcripts may engage translocon-bound ribosomes for closer membrane proximity (18). In addition, certain affected transmembrane proteins translocate from the ER to the mitochondria, thereby revealing a potential role of RRBP1 in cellular intercompartmental communication (15). Furthermore, yeast two-hybrid analyses have indicated that the C-terminal region of RRBP1 interacts with the kinesin family member 5B (KIF5B) (19), which has markedly high expression in a variety of cancer cell lines (13), such as MCF-7, HeLa and U2OS cells (20). RRBP1, a receptor for KIF5B, is involved in vesicle transport or mRNA localization of ER origin and its role of combining ribosomes with kinesins suggests its potential involvement in tumor transport or tumor cell invasion processes.

### Roles of RRBP1 in organelle interactions

Mitochondria-ER contacts (MERCs), which serve as an interface for communication between ER and mitochondria, are involved in regulating mitochondrial energy metabolism homeostasis, membrane phospholipid remodeling, programmed cell death signaling and other core biological processes by mediating calcium-ion dynamic homeostasis and the exchange of intermediates in lipid metabolism (21). Abnormal function of MERCs can interfere with the synergistic network of cellular organelles and thus lead to tumorigenesis, neurodegenerative diseases and cardiovascular system dysfunction (21); therefore, MERCs might have potential value as targets for disease intervention. RRBP1 mediates MERC formation through a specific transmembrane interaction between its PDZ-binding domain and the PDZ domain of the mitochondrial protein synaptojanin-2 binding protein (SYNJ2BP). This interaction notably regulates mitochondrial DNA replication and spatial organization, thereby modulating mitochondrial genomic stability and functional dynamics (14,21,22). Protein translation inhibitors such as puromycin disrupt the RRBP1-SYNJ2BP interaction and specifically eliminate mitochondrial contacts with ribosome-enriched rough ER (14). Knockdown of RRBP1 almost completely eliminates ribosome-rich rough ER contact sites (riboMERCs), leaving only certain smooth-type contact sites and leads to a decrease in mitochondrial membrane potential; therefore, RRBP1 appears to be essential for mitochondrial function (21). Glycoprotein (Gp)78 ubiquitin ligase activity regulates the size and tubular morphology of riboMERCs. Gp78 upregulation induces the formation of extended riboMERCs in COS-7 and HeLa cells in the presence of RRBP1 (21). This mechanism suggests that RRBP1 provides a structural basis and Gp78 finely regulates the complexity of the contact sites through a ubiquitination-dependent pathway. In addition, dynamin-related protein 1 deficiency disrupts RRBP1-SYNJ2BP interactions and leads to defects in mitochondrial DNA distribution (22). Dynamin-related protein 1 deficiency indirectly affects ER lamellar structure and RRBP1 function by interfering with mitochondrial division dynamics (22), thus suggesting a key role of ER-mitochondrial synergistic interactions in mitochondrial genome regulation.

Furthermore, the ER-mitochondrial contact site also serves a key role in mitochondrial autophagy. RRBP1 mediates selective mitophagy induced by mitochondrial protein import stress (MPIS) through dynamic subcellular relocalization (perinuclear rough ER to peripheral ER). Under MPIS conditions, the cytosolic-retained Nod-like receptor protein nucleotide-binding, leucine-rich repeat X1 forms a complex with RRBP1 and drives microtubule-associated protein 1 light chain 3 lipidation and autophagosome formation. This process is independent of the canonical phopsphatase and tensin homolog-induced putative kinase 1 (PINK1)-parkin RBR E3 ubiquitin protein ligase (PRKN) pathway and preferentially uses ER-mitochondria contact sites as membrane sources (23,24). RRBP1 exhibits dual functionality; it assists in localized translational recovery during acute stress but switches to a pro-autophagic mode under prolonged stress. The interaction between RRBP1 and splicing factor, proline- and glutamine-rich, suggests a potential role of RRBP1 in regulating the subcellular localization of mitochondrially-associated mRNAs during stress recovery (23). Notably, PINK1 and PRKN are genetic risk factors for Parkinson's disease and accumulating evidence suggests that mitochondrial quality control defects are a key molecular basis for neurodegeneration (24–27). Consistent with this possibility, Sierksma *et al* (28) have identified *RRBP1* as a subthreshold Alzheimer's disease risk gene. In parallel with its stress-adaptive function, RRBP1 modulates ER ribosome interactions in neurons. RRBP1 is enriched in the ER tubules of neuronal axons and is virtually absent in dendrites. Nerve growth factor neurotrophin-3 specifically enhances ER-ribosome interactions through RRBP1, whereas brain-derived neurotrophic factor/nerve growth factor has no such effect, thus suggesting selective regulation of the signaling pathway (29). In addition, the identification of RRBP1 in the neuromuscular junction has confirmed its specific distribution in the synaptic region. RRBP1 may be involved in the localization of synaptic proteins by binding the kinesin KIF5B, which might serve as a novel candidate target for exploring the mechanisms of neuromuscular diseases (30). Whether mediating MPIS-induced mitochondrial autophagy or regulating axonal ER-ribosome dynamics, RRBP1 functions as a sub-stable hub at the organelle-membrane interface.

### Roles of RRBP1 in lipid metabolism

*RRBP1* has been identified as a key gene in the regulation of lipid levels in an integrated multi-omics study (31). Observations that RRBP1 expression quantitative trait loci markedly co-localize with genetic association signals for low-density lipoprotein, total cholesterol and non-high-density lipoprotein suggest that *RRBP1* is an effector gene for lipid-related genetic variation (31). RRBP1 is markedly upregulated in adipose stem and progenitor cells in the subcutaneous adipose tissue of mice with obesity induced by a high-fat diet (32). In addition, a cellular compartment in hepatocytes, denoted wrappER, has been identified to functionally and structurally integrate all hepatocyte systems and intracellular fatty acid elimination pathways (33). When RRBP1 expression is inhibited in the liver, the uniform juxtaposition between wrappER and mitochondria is disrupted and localized abnormal bumps form (33). When the complex comprising RRBP1 and SYNJ2BP is not functional, the directional flow of fatty acids from the ER to the mitochondria is impeded and lipids abnormally accumulate in liver cells; therefore, the RRBP1-SYNJ2BP axis directly influences the balance of liver lipid stability by modulating the physical properties of the membrane-contact interface (33). In parallel, RRBP1 silencing decreases very low-density lipoprotein biosynthesis and triggers intrahepatic triglyceride and free fatty acid accumulation, which in turn markedly increases lipid droplet accumulation and the expression level of the lipid droplet marker perilipin 2 (34). These findings demonstrated that RRBP1 is important in regulating hepatic lipid metabolic homeostasis by maintaining the structural integrity of the contact between the ER and mitochondria.

## 3. Roles of RRBP1 in various types of cancer

**Figure 2.**
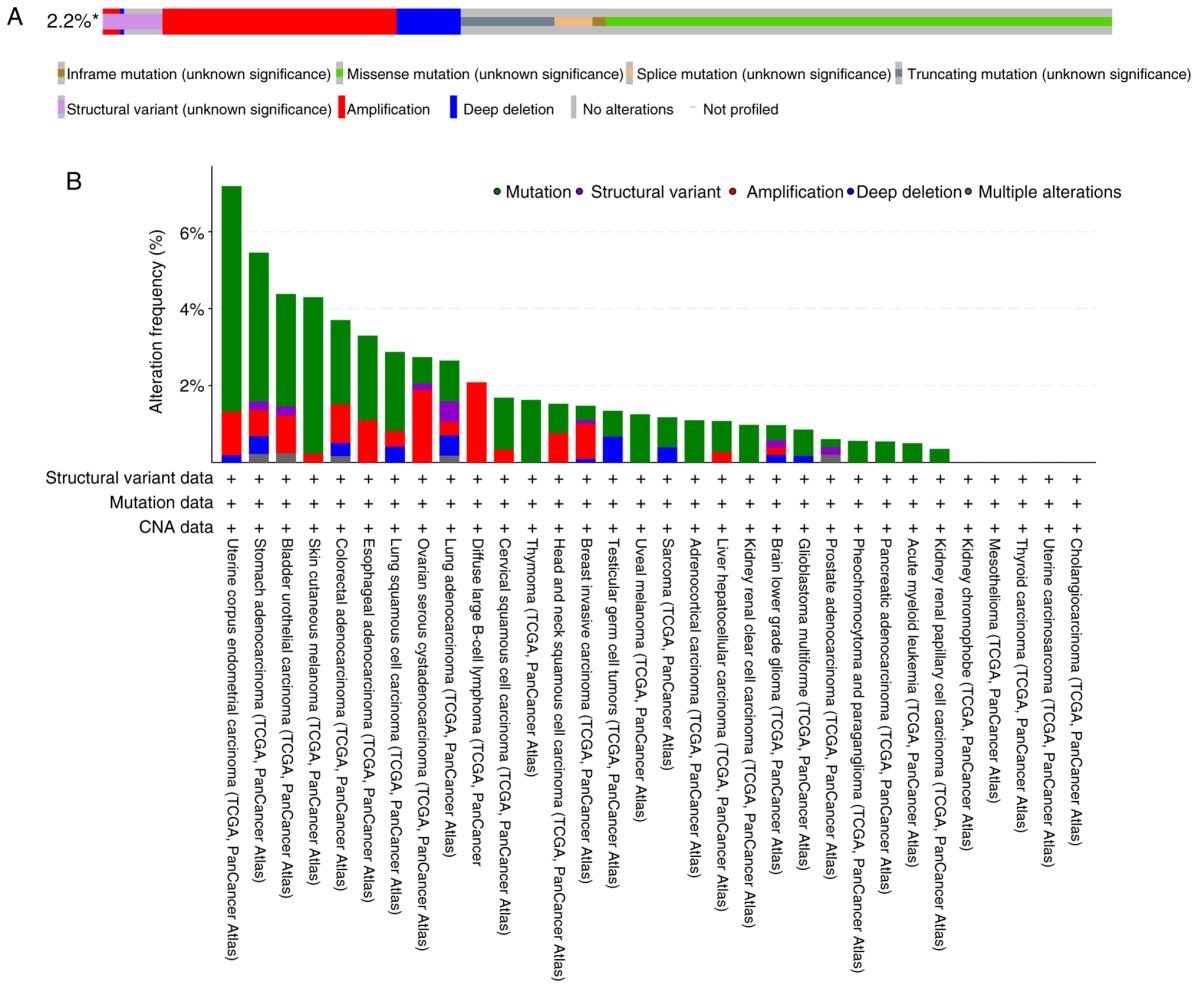
Pan-cancer landscape of RRBP1 genomic alterations (cBioPortal database). (A) RRBP1 gene OncoPrint visualization. An asterisk (*) indicates that not all samples are profiled. (B) RRBP1 gene mutation overview in various cancer types. CNA, copy number alteration; TCGA, The Cancer Genome Atlas; RRBP1, ribosome-binding protein 1.

RRBP1 serves important regulatory roles in cancer genesis and evolution. Due to its role in organelle interactions, its molecular mechanisms and pathological associations are receiving increasing attention (8,9,35–37). RRBP1 influences tumor metabolic reprogramming and microenvironmental adaptation by dynamically regulating substance transport and signaling between subcellular compartments and therefore might have translational value as a potential therapeutic target. To comprehensively evaluate the contributions of genetic alterations to RRBP1-driven oncogenesis, the present review characterized the oncogenic status of RRBP1 across multiple cancer types using the cBioPortal database for Cancer Genomics platform (https://www.cbioportal.org/) (Fig. 2). Based on The Cancer Genome Atlas (TCGA) Pan-Cancer Atlas studies, the present review generated an OncoPrint visualization map depicting the types and occurrence frequencies of *RRBP1* genetic alterations, while also illustrating the lineage distribution characteristics of specific *RRBP1* mutations. Among 10,967 samples, 237 samples exhibited *RRBP1* alterations, accounting for ~2.2% of all cases (Fig. 2A). These alterations were primarily missense mutations. Among these, uterine corpus endometrial carcinoma, stomach adenocarcinoma, bladder urothelial carcinoma and skin cutaneous melanoma exhibited relatively high alteration frequencies (>4%) (Fig. 2B). By contrast, because the *RRBP1* gene in cholangiocarcinoma, uterine carcinosarcoma, thyroid carcinoma, mesothelioma and kidney chromophobe demonstrated no changes (Fig. 2B), the oncogenic role of RRBP1 in these cancer types may be limited.

The following section discusses research progress on RRBP1 in tumors and explores the precision therapeutic potential of targeting RRBP1 or its microenvironmental adaptors according to its multiple regulatory mechanisms.

### Lung cancer

**Figure 3.**
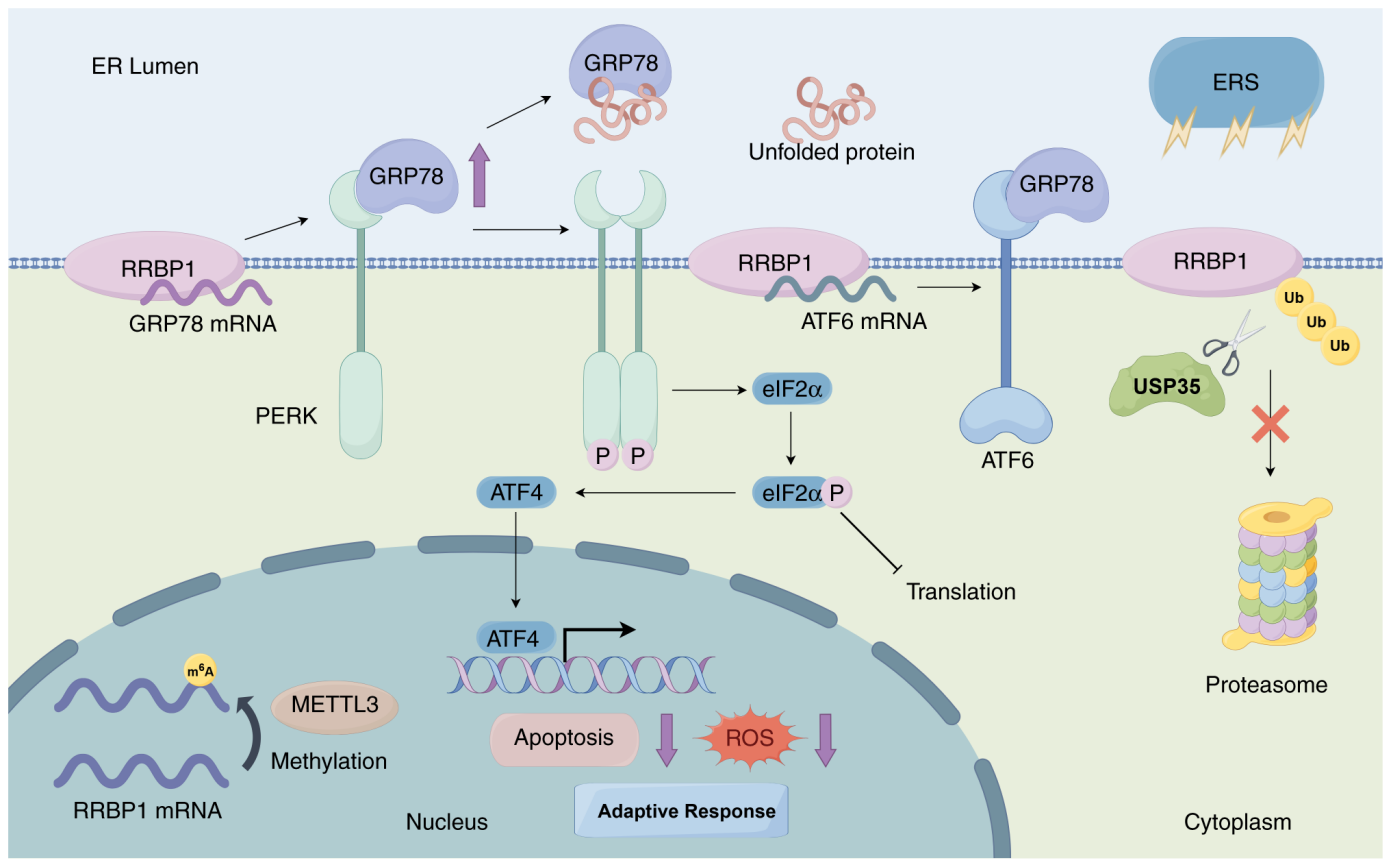
RRBP1 adapts to ERS through the UPR and enhancement of self-stabilization. RRBP1 upregulates GRP78 expression by stabilizing GRP78 mRNA and regulates ATF6 mRNA to stabilize ATF6 for participation in the UPR. In addition, RRBP1 inhibits degradation pathways through METTL3-mediated m6A methylation and USP35-mediated deubiquitination, thus enhancing self-stability and promoting adaptive stress signaling. RRBP1, ribosome-binding protein 1; Ub, ubiquitin; UPR, unfolded protein response; ERS, endoplasmic reticulum stress; GRP78, glucose-regulated protein 78; ROS, reactive oxygen species; METTL3, methyltransferase-like 3; USP35, ubiquitin-specific processing protease 35; ATF6, activating transcription factor 6; m6A, N6-methyladenine; PERK, protein kinase R-like endoplasmic reticulum kinase.

A previous study reported that several mutated genes, including *RRBP1*, have been identified to be enriched primarily in nucleoplasmic localization and DNA repair-related biological processes (38) and therefore, may be involved in genetic susceptibility to lung adenocarcinoma by influencing the DNA damage response mechanism. In addition, RRBP1 is markedly upregulated in lung cancer tissues and its upregulation is associated with early tumor stages (36). RRBP1 is also associated with the unfolded protein response (UPR), a central mechanism through which tumor cells respond to ER stress (ERS) (36). Cells rely on the ER to properly fold and process secreted proteins and membrane proteins; however, when protein folding is disrupted by various physiological or pathological stimuli, such as deficient autophagy, energy deprivation, inflammatory challenges and hypoxia, misfolded or unfolded proteins accumulate, in the condition known as ERS (39). To restore ER homeostasis, cells activate an adaptive mechanism, the UPR, which is mediated by three classic ER-resident sensors: Inositol-requiring enzyme 1α, protein kinase R-like endoplasmic reticulum kinase (PERK) and activating transcription factor 6 (ATF6) (40). The UPR decreases global protein synthesis, enhances the production of molecular chaperones and promotes the degradation of misfolded proteins, thereby alleviating the burden on the ER (40). Although the UPR is initially protective, a prolonged or unresolved UPR may trigger apoptotic signals, thus serving dual roles in cell survival and death. Tumor cells undergo persistent ERS because of rapid proliferation and a harsh microenvironment and must rely on the UPR to maintain their survival. Glucose-regulated protein 78 (GRP78), a regulator of the UPR, is markedly upregulated in lung cancer. However, when GRP78 is upregulated, it dissociates from the UPR signaling sensor PERK; therefore, PERK dimerizes and undergoes autophosphorylation (36,41). Phosphorylated PERK further catalyzes the phosphorylation of eukaryotic translation initiation factor eukaryotic initiation factor 2α (eIF2α), thus leading to translational repression, a core adaptive strategy enabling cells to cope with mild ERS (Fig. 3) (36,41). Tsai *et al* (36) have demonstrated that knockdown of RRBP1 exacerbates ERS and markedly decreases cell viability and tumor formation. This knockdown also markedly decreases ATF6 mRNA levels; therefore, RRBP1 might be involved in the UPR by regulating the mRNA stability of ATF6 (Fig. 3) (36). By contrast, upregulation of RRBP1 enhances the anti-apoptotic ability of tumor cells. RRBP1 protects cells against ERS-induced apoptosis by upregulating the expression level of GRP78 and simultaneously decreasing the accumulation of reactive oxygen species (ROS) (36). Therefore, RRBP1 regulates the UPR by maintaining the mRNA stability of GRP78, which in turn helps lung cancer cells adapt to ERS and chemotherapeutic stress and maintains tumor survival and progression. Furthermore, in non-small cell lung cancer, elevated expression of ubiquitin-specific processing protease 35 (USP35) markedly upregulates RRBP1 protein levels (41). USP35 stabilizes RRBP1 protein by directly binding RRBP1 and catalyzing its deubiquitination modification, thereby inhibiting the proteasome-mediated degradation pathway (Fig. 3) (41). The USP35/RRBP1 axis exerts a protective effect through a dual mechanism; it enhances GRP78 expression and PERK phosphorylation levels (adaptive UPR signaling) but also inhibits the CHOP-caspase3 apoptotic pathway, thus markedly decreasing apoptosis (41).

### Breast cancer

The mRNA and protein expression levels of RRBP1 in breast cancer tissues are markedly higher compared with those in normal tissues (4). RRBP1 expression is markedly associated with breast cancer histological grade, human EGFR 2 (HER-2) status, p53 status and molecular subtypes; furthermore, in patients with HER-2^+^ breast cancer, increased RRBP1 expression is associated with worse overall survival (OS) (4). The upregulation of RRBP1 protein localizes primarily in the perinuclear region of the cytoplasm (13). Normal breast tissues demonstrate only weak to moderate cytoplasmic staining, whereas the staining intensity is markedly enhanced in cancerous tissues. RRBP1 expression is higher in malignant breast cancer types compared with that in benign or proliferative lesions; however, no gradient in RRBP1 expression has been observed in benign or proliferative conditions (13). RRBP1 expression and lymph node metastasis (LNM) are independent prognostic factors for OS in patients with HER-2^+^ breast cancer, according to a multivariate analysis (4). RRBP1 upregulation markedly influences survival in patients with early-stage breast cancer, but this association has not been observed in patients with advanced-stage types of cancer (4). RRBP1 promotes breast cancer cell survival by participating in the UPR and this mechanism might underlie its association with poor prognoses in patients with HER-2^+^ breast cancer (4).

### Liver cancer

In hepatocellular carcinoma (HCC) studies, RRBP1 has been shown to serve a key role in high glucose-mediated tumor malignant progression through the E2F transcription factor 1 (E2F1)/RRBP1 signaling pathway (42). He *et al* (42) have reported that high glucose treatment markedly upregulates the mRNA and protein expression levels of RRBP1 in HepG2 cells, whereas knockdown of RRBP1 markedly inhibits cell proliferation, migration and invasive ability. RRBP1 has been suggested to be a core molecule mediating the pro-cancer effect of high glucose. Furthermore, RRBP1 expression under cellular stress conditions relies on an internal ribosome entry site (IRES)-mediated translation mechanism (43). IRES elements are structured RNA motifs within the 5′-untranslated regions of select mRNAs that enable cap-independent initiation of translation (44). Under stress conditions, when canonical cap-dependent protein synthesis is compromised, IRES elements directly recruit the 40S ribosomal subunit, often with assistance from IRES trans-acting factors, thereby sustaining the translation of specific transcripts key for cell survival, adaptation and reprogramming (44). Notably, in cancer, dysregulated IRES-mediated translation and IRES trans-acting factor activity have been implicated in tumorigenesis and therapeutic resistance (45). The 5′-untranslated region of RRBP1 mRNA has IRES activity, which is markedly enhanced under chemotherapeutic drug treatment or serum starvation (43). The La autoantigen facilitates ribosome recruitment by binding the core region of the IRES (−237 to −58 nt), whose two Gdf5 regulatory region loop structures are essential for La protein binding (43). In human HCC cells, RRBP1 protein levels are elevated under the aforementioned stress conditions, but its mRNA transcripts do not markedly change, thus suggesting that the enhancement of translational efficiency originates from an IRES-dependent mechanism (43). Particularly, chemotherapeutic drugs activate IRES-mediated translation by upregulating the expression level of La protein, whereas serum starvation induces La protein translocation from the nucleus to the cytoplasm, both of which work together in activating IRES-mediated translation (43).

In summary, RRBP1 drives tumor malignant progression in high glucose environments through the E2F1/RRBP1 signaling axis and E2F1 directly binds RRBP1, and enhances its transcriptional activity, thereby promoting HCC cell proliferation, migration and invasion. Under chemotherapeutic or serum starvation conditions, RRBP1 enhances the efficiency of protein synthesis through an IRES-mediated translation mechanism, which in turn enhances the survival of HCC cells in adverse conditions. This mechanism does not depend on changes in mRNA transcript levels but instead relies on translation regulation to achieve rapid stress adaptation.

### Colorectal cancer

**Figure 4.**
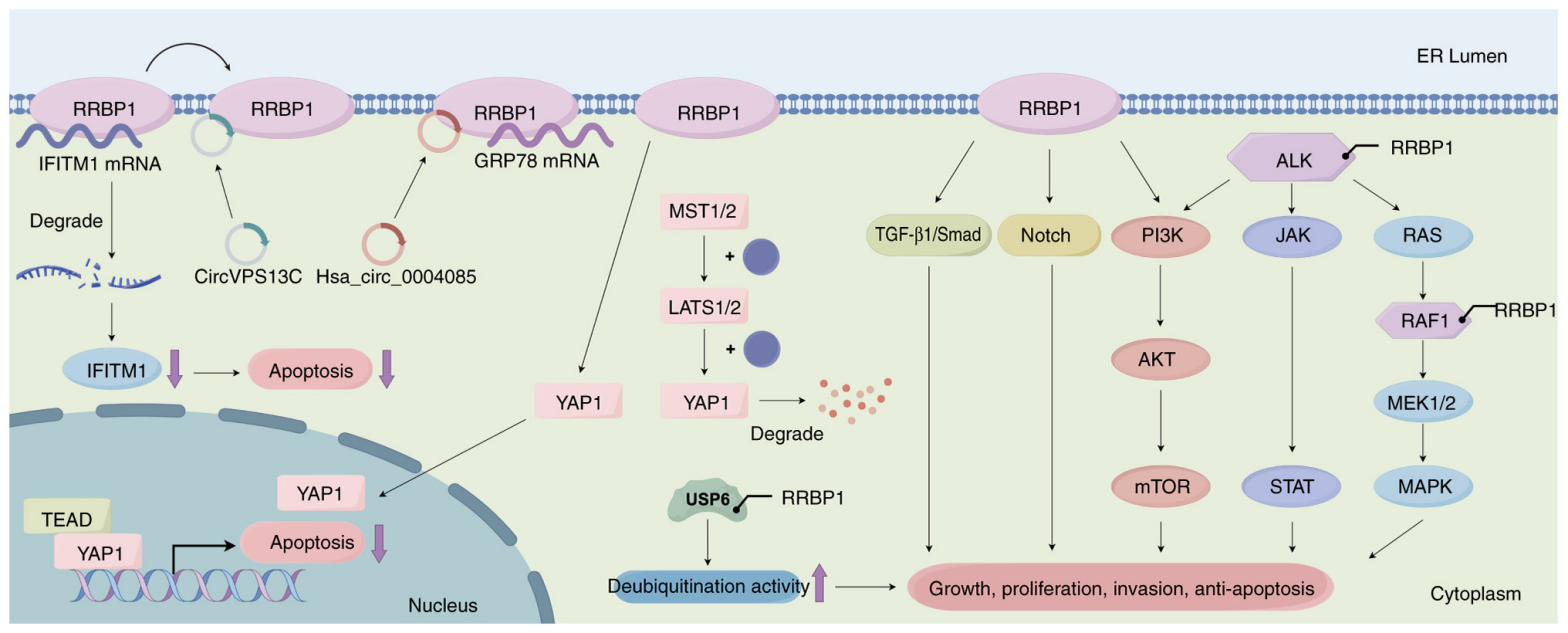
RRBP1 promotes anti-apoptosis through different signaling pathways, gene fusions and interactions with circRNAs. RRBP1 activates various signaling pathways, thereby promoting tumor cell proliferation and anti-apoptosis. It also inactivates Hippo signaling and activates pro-survival and anti-apoptotic genes. In addition, *RRBP1* activates kinase function through gene fusion (*ALK, RAF1* and *USP6*), promotes MAPK and other pathways and upregulates deubiquitination. Furthermore, in the interaction between RRBP1 and circRNAs, binding of RRBP1 to hsa_circ_0004085 stabilizes GRP78 and enhances cellular adaptation to ERS. By contrast, circVPS13C inhibits the binding of RRBP1 to IFITM1 mRNA by competitively binding RRBP1; subsequently, degradation of IFITM1 mRNA leads to notable downregulation of its protein expression level and further inhibits apoptosis. RRBP1, ribosome-binding protein 1; YAP1, Yes-associated protein 1; ALK, anaplastic lymphoma kinase; GRP78, glucose-regulated protein 78; ERS, endoplasmic reticulum stress; IFITM1, interferon-induced transmembrane protein 1; circRNA, circular RNA; hsa, *Homo sapiens*; TEAD, transcription enhancement associated domain family members; MST1/2, mammalian sterile 20-like kinase 1/2; LATS1/2, large tumor suppressor 1/2.

RRBP1 mRNA is higher in colorectal cancer (CRC) tissues compared with normal tissues and RRBP1 protein is less variable in normal tissues but more heterogeneous in cancerous tissues (5). RRBP1 expression is predictive of unfavorable survival outcomes; patients with high RRBP1 expression have shorter disease-specific survival (5). The positive association between chromosomal gains and RRBP1 mRNA levels (5) suggests the potential involvement of *RRBP1* in CRC progression as a driver gene. RRBP1 is also associated with chemoresistance in CRC. The circular RNA (circRNA) hsa_circ_0004085 is a key molecule mediating chemoresistance in *Fusobacterium nucleatum*-infected colon cancer cells (46). Furthermore, RRBP1 is a direct binding protein of hsa_circ_0004085 (46). Hsa_circ_0004085 inhibits chemotherapeutic drug-induced ERS-associated apoptosis by enhancing the stability of GRP78 mRNA by binding RRBP1 and upregulating the expression level of GRP78, thus promoting chemoresistance in tumor cells (Fig. 4) (46). Furthermore, hsa_circ_0004085 activates the UPR by promoting the translocation of the sheared form of ATF6 (ATF6p50) to the nucleus, thereby enhancing the adaptive survival of tumor cells (46).

*RRBP1* is involved in tumor progression, has utility as a prognostic marker, is a driver gene in CRC and its expression level is also closely associated with chromosomal abnormalities. Furthermore, it regulates chemoresistance-related pathways through interaction with hsa_circ_0004085. Therefore, CRC treatments should integrate the role of RRBP1 at the molecular and cellular levels to overcome the associated resistance mechanisms.

### Bladder cancer and upper tract urothelial carcinoma (UTUC)

The mRNA and protein expression levels of RRBP1 in bladder cancer tissues are markedly higher compared with that in normal bladder tissues (6). RRBP1 is highly expressed in advanced-stage, LNM and basal squamous subtype bladder cancer and is closely associated with poor prognosis in patients (6–8). In addition, Luo *et al* (8) have identified that the *RRBP1* gene region exhibits aberrant DNA methylation in UTUC, according to methylation profiling microarray analysis, and its mRNA and protein expression levels are upregulated in both tumor tissues and a UTUC cell line. Experimental overexpression of RRBP1 has been found to promote the proliferation of bladder cancer cells, whereas downregulation of RRBP1 inhibits cell proliferation (6). This proliferative effect is associated with the TGF-β1/SMAD signaling pathway, which is activated by RRBP1 upregulation (Fig. 4) (6). Wang *et al* (7) have reported that knockdown of RRBP1 results in the downregulation of C-C chemokine receptor type 7 and subsequent inhibition of the migration and invasive ability of bladder cancer cells. In addition, knockdown of RRBP1 inhibits the proliferation, migration and invasive ability of UTUC cells (8). The high expression level of RRBP1 drives the malignant progression of tumors through a multidimensional regulatory network. Molecular subtyping analysis has revealed that the high expression level of RRBP1 is positively associated with genes related to cell proliferation; therefore, it might promote cell cycle progression through activation of the PI3K/AKT and Notch signaling pathways (Fig. 4). Furthermore, RRBP1 upregulates epithelial-mesenchymal transition (EMT) genes and basal cell markers and suppresses luminal markers, thus contributing to the acquisition of migratory and invasive properties of tumor cells and a stem cell-like drug-resistant phenotype (8). The EMT is a reversible cellular program in which epithelial cells lose their polarity and intercellular adhesion through the downregulation of epithelial markers such as E-cadherin and concurrently acquire mesenchymal characteristics, including increased motility and the upregulation of N-cadherin and vimentin (47). This phenotypic shift facilitates tumor cell invasion through the basement membrane and entry into the circulatory or lymphatic systems, thus enabling metastatic dissemination. Notably, EMT is closely associated with the acquisition of cancer stem cell-like traits, including enhanced survival ability, resistance to apoptosis and increased expression levels of drug efflux transporters (47). These features collectively contribute to resistance against chemotherapy and immunotherapy. Furthermore, in a UTUC patient-derived organoid model, high expression levels of RRBP1 have been associated with chemoresistance to cisplatin, gemcitabine and epirubicin (8); therefore, it synergistically promotes tumor cell survival by modulating ERS and yes-associated protein 1 (YAP1) signaling (Fig. 4) (8).

Overall, RRBP1 hypomethylation drives its upregulation and subsequent participation in the malignant progression of UTUC by promoting cell proliferation, the EMT process and chemoresistance. Therefore, targeting RRBP1 or its downstream effector pathways can reverse malignant tumor phenotypes. In bladder cancer, RRBP1 serves a role in cell proliferation through the TGF-β1/SMAD signaling pathway, whereas cell migration and invasion involve the regulation of C-C chemokine receptor type 7 and ERS, all of which may serve as potential biomarkers for diagnosis and prognostic evaluation, thus providing a precise therapeutic strategy for patients with high RRBP1 expression.

### Prostate cancer

The mRNA and protein expression levels of RRBP1 are higher in prostate cancer tissues compared with those in normal cancer-adjacent tissues (9). Patients with high (≥8) Gleason scores have higher RRBP1 expression levels in tumor tissues compared with low Gleason scores (9). Furthermore, RRBP1 expression is associated with the T stage of prostate cancer, LNM and prostate-specific antigen level (9,48). The median survival time is shorter in patients with prostate cancer with higher compared with lower RRBP1 expression. Multifactorial Cox regression analysis has indicated that RRBP1 is an independent prognostic factor (9,48). Therefore, RRBP1 is closely associated with tumor malignancy and progression and high RRBP1 expression can serve as a diagnostic marker and a predictor of poor prognosis in prostate cancer. Methyltransferase-like 3 (METTL3), an RNA methyltransferase that forms a core methyltransferase complex with METTL14, is responsible for adding N6-methyladenine (m^6^A) modifications, which is the most common internal modification in eukaryotic RNAs (49,50). Rather than functioning through a single pathway, m^6^A modifications influence multiple RNA processes by recruiting reader proteins such as YT521-B homology m^6^A RNA binding protein F2, which recognize m^6^A-marked transcripts and promote their degradation (51). For example, m^6^A and its reader proteins have been shown to regulate multiple steps of RNA metabolism, including alternative pre-mRNA splicing (via the nuclear reader YTHDC1) (52), mRNA stability and degradation (via YTHDF2) (51), and translation efficiency through enhanced ribosome loading (via YTHDF1) (53). These findings supported that METTL3-mediated m^6^A modification is a dynamic epitranscriptomic mechanism shaping mRNA fate and cellular function. Notably, abnormal METTL3 expression and the resulting imbalance in m^6^A regulation have been associated with pathological conditions, including tumorigenesis (54). *RRBP1* is a key downstream target gene regulated by METTL3 in an m^6^A-dependent manner. METTL3 upregulates the expression level of RRBP1 by binding and methylating RRBP1 mRNA and enhancing its stability (Fig. 3) (49). Furthermore, m^6^A-sequencing has revealed that RRBP1 mRNA demonstrates stronger m^6^A enrichment in tumor tissues compared with non-tumor tissues, thus further supporting the molecular mechanism of its regulation by METTL3 (49).

In summary, RRBP1 is a core effector molecule of the METTL3/m^6^A signaling axis in prostate cancer and its m^6^A-dependent stabilization promotes tumor aggressiveness and is closely associated with poor patient prognosis. Peptide inhibitors targeting this regulatory axis have potential therapeutic value in the future.

### Epithelioid inflammatory myofibroblastic sarcoma (EIMS)

EIMS is a rare inflammatory myofibroblastic tumor (IMT) subtype. IMT rarely recurs or metastasizes, whereas the epithelioid variant of IMT, EIMS, which has distinctive morphological features-for example, loosely arrayed round to epithelioid tumor cells with vesicular nuclei, prominent nucleoli, eosinophilic to amphophilic cytoplasm (55) and clinical aggressiveness, exhibits poor prognosis (56,57). Chromosomal rearrangements are frequently observed in IMT, including *ALK* rearrangement, a genetic abnormality driving tumor cell proliferation in the form of fusions of various chaperone genes such as *TPM3-ALK, TPM4-ALK, CLTC-ALK* and *EML4-ALK* (56,58); furthermore, the diversity of recombination patterns may be closely associated with clinical heterogeneity (58). Notably, EIMS usually exhibits nuclear membrane localization of Ran binding protein 2-ALK fusion oncoproteins; in addition, novel RRBP1-ALK fusion proteins have been identified in aggressive EIMS cases, showing cytoplasmic ALK expression with perinuclear accentuation (Fig. 4) (56). The molecular structure and 3D simulation structure of the RRBP1-ALK fusion protein are shown in Fig. S1. The protein sequence of RRBP1-ALK was obtained from the Ensembl database (https://www.ensembl.org/, release 114) and its domain architecture was annotated using the Simple Modular Architecture Research Tool (SMART; http://smart.embl.de/, accessed 12 August 2025). A three-dimensional structural model was subsequently generated with the AlphaFold server (https://alphafoldserver.com/, accessed 12 August 2025). Each ALK fusion partner retains a coiled-coil structural domain in the ALK fusion protein, whereas RRBP1-ALK combines the RRBP1 coiled-coil structural domain, the RRBP1 ER transmembrane structural domain and the ribosome receptor structural domain (56). The mechanism of the formation of this fusion gene involves aberrant splicing of intron 20 of the *RRBP1* gene; therefore, the *RRBP1-ALK* open reading frame is composed of a 33-nucleotide sequence of *RRBP1* exon 20 fused to *RRBP1* intron 20, which in turn is fused to *ALK* exon 20 (56). This fusion pattern results in an ALK protein with a cytoplasmic localization and an enhanced perinuclear expression profile (56). EIMS cases with *RRBP1-ALK* fusion exhibit a rapidly progressive and aggressive disease course, with a median survival of only 2–10 months, which is markedly shorter compared with that of spindle cell inflammatory myofibroblastoma with conventional *ALK* fusion (56). Lee *et al* (56) have suggested that *RRBP1-ALK* is a novel recurrent oncogenic mechanism in clinically aggressive EIMS. Although previous reports indicated that the *RRBP1-ALK* fusion gene is seen only in EIMS, subsequent reports have described the *RRBP1-ALK* fusion gene in IMT (58,59). In a case report of recurrent IMT, the 3′-end of exon 19 of the *ALK* gene was fused to the 5′-end of exon 10 of the *RRBP1* gene (58). This case histologically revealed a spindle cell type IMT, which differed from the morphological features and clinical behavior of EIMS (58). Therefore, the *RRBP1-ALK* fusion might have a heterogeneous pathogenic mechanism in different IMT subtypes. A separate report presented a case with a characteristic strong perinuclear ALK^+^ expression pattern, in agreement with the expression profile of previously reported cases of *RRBP1-ALK* fusion (56), and consisted of loosely organized spindle cells on a mucus-like background (59). Furthermore, a recent study has identified an *RRBP1-USP6* fusion in low-grade myofibroblastic sarcoma (Fig. 4) (60). This fusion arises from a T (17;20) chromosomal translocation that juxtaposes exon 1 of the *USP6* gene with exon 2 of the *RRBP1* gene, thus marking the first instance of *RRBP1* being characterized as a fusion partner for *USP6* (60). Structurally, this implies that the fusion transcript retains the full coding sequence of *USP6* together with its key functional domains, including the ubiquitin-specific protease catalytic core. *RRBP1* functions as a regulatory module, driving the abnormal expression of USP6. This finding has expanded the genetic spectrum of USP6-associated neoplasms and is potentially further links ER-ribosome pathway dysregulation and malignant transformation in myofibroblastic tumors.

In conclusion, *RRBP1* drives tumor malignancy through fusion with *ALK* or *USP6* genes and the *RRBP1-ALK* fusion might enhance cancer cell survival and invasiveness through ER localization, whereas the *RRBP1-USP6* fusion aberrantly activates ubiquitination regulation, thus suggesting an association between ER-ribosome pathway dysregulation and tumor progression.

### Pancreatic cancer

*RRBP1* forms a fusion with the *Raf1* gene (*RRBP1-Raf1*), which is markedly enriched in *KRAS* wild-type tumors, according to the Arriba algorithm developed by Uhrig *et al* (61). This fusion retains the intact coding sequence of the Raf1 kinase domain, whereas the transmembrane domain of RRBP1 anchors Raf1 to the ER and drives its constitutive activation (61). Based on the structural features described, it can be inferred that the breakpoint of the RRBP1-Raf1 fusion protein is located near the C-terminus of RRBP1, thereby preserving its functional N-terminal domain and within the N-terminal regulatory region of Raf1, maintaining the integrity of its kinase domain. The structural preservation of the key domains of both partners implies a breakpoint that occurs after the functional segments of RRBP1 and before the key serine/threonine kinase domain of Raf1, possibly resulting in a reading frame that maintains the open reading frame and functional activity of the fusion oncoprotein. Furthermore, functional studies demonstrated that RRBP1-Raf1 expression in *TP53*-deficient MCF10A cells and pancreatic ductal epithelial H6c7 cells markedly enhances EGF-independent colony formation (61). After EGF withdrawal, the fusion protein sustains MAPK pathway activation, as evidenced by markedly elevated phosphorylation levels of MEK1/2 and ERK1/2 (Fig. 4) (61). In brief, the preservation of the Raf1 kinase domain in this fusion might confer differential sensitivity to specific Raf inhibitors. This finding expands the molecular mechanisms underlying MAPK pathway hyperactivation in pancreatic cancer and provides a novel therapeutic direction for *KRAS* wild-type pancreatic cancer.

### Oral squamous cell carcinoma (OSCC)

RRBP1 is markedly upregulated in both early-stage and late-stage cisplatin-resistant OSCC cell lines, as well as in tumor tissues from chemotherapy-non-responsive patients vs. those with chemotherapy-sensitive disease (62). Mechanistically, RRBP1 mediates chemoresistance by regulating the Hippo signaling pathway effector YAP1 (Fig. 4) (62). The Hippo signaling pathway, governed by mammalian sterile 20-like kinase 1/2 and large tumor suppressor (LATS) 1/2 kinases, is implicated in cancer chemoresistance. After activation, LATS1/2 phosphorylates key residues of YAP1 and transcriptional coactivator with PDZ-binding motif (TAZ) [YAP1 serine (Ser) 127 and Ser397], thus leading to their cytoplasmic retention and subsequent degradation via the β-transducin repeat-containing protein-dependent ubiquitin-proteasome system (62). By contrast, Hippo pathway inactivation results in dephosphorylated YAP1/TAZ evading degradation, translocating to the nucleus, binding transcription enhancement-associated domain family members (TEADs) and activating pro-survival and anti-apoptotic genes (62). Notably, RRBP1 expression is strongly associated with YAP1 target genes and RRBP1 knockdown markedly decreases YAP1 and its downstream targets at both the mRNA and protein levels (62). Furthermore, RRBP1 depletion restores cisplatin sensitivity and enhances apoptosis, as evidenced by increased expression levels of cleaved PARP (62). Therefore, these findings established RRBP1 as a central driver of cisplatin resistance in OSCC, through a mechanism involving Hippo pathway dysregulation, and highlight RRBP1 targeting as a therapeutic strategy to potentially overcome cisplatin-induced chemoresistance in advanced OSCC in the future.

### Pituitary adenoma

In non-functioning pituitary adenomas (NFPAs), the function of RRBP1 is uniquely modulated by the circRNA circVPS13C and demonstrates a distinct regulatory mechanism (63). CircVPS13C competitively binds RRBP1 and disrupts its interaction with interferon-induced transmembrane protein 1 (IFITM1) mRNA, thereby decreasing IFITM1 mRNA stability and suppressing its expression (Fig. 4) (63). IFITM1, which is known to regulate cell proliferation and migration, exerts tumor-suppressive effects in pituitary tumors; overexpression of IFITM1 markedly inhibits the proliferation of pituitary tumor-derived folliculostellate cells and induces apoptosis (63). Zhang *et al* (63) have revealed that RRBP1 silencing markedly decreases IFITM1 mRNA and protein levels, whereas overexpression of RRBP1 partially rescues the inhibitory effects of circVPS13C on IFITM1. In contrast to its pro-tumorigenic roles in other cancer types, such as lung cancer, bladder cancer and EIMS, RRBP1 exhibits tissue-specific regulation in NFPAs by sustaining the tumor-suppressive function of IFITM1 (63). This finding underscores the functional diversity of RRBP1 in tumor microenvironments and highlights the key regulatory role of the circVPS13C-RRBP1-IFITM1 axis in NFPA progression.

### Other cancer types

In female reproductive system cancer types, RRBP1 exhibits marked upregulation with distinct clinicopathological associations. For instance, in endometrial carcinoma, elevated RRBP1 levels are strongly associated with advanced International Federation of Gynecology and Obstetrics (FIGO) stages, poor differentiation, deep myometrial invasion and LNM (37). Similarly, cervical squamous cell carcinoma and epithelial ovarian cancer demonstrate markedly higher RRBP1 expression in tumor tissues compared with normal tissues and this elevated expression is associated with aggressive features such as advanced FIGO staging, lymph node involvement and unfavorable histotypes (35,64). Notably, multivariate Cox analyses across these malignancies have consistently identified RRBP1 as an independent prognostic factor whose high expression predicts shortened OS and disease-free survival (35,37,64).

In acute myeloid leukemia (AML), RRBP1 has been reported as a potential leukemogenesis-associated molecule regulated by exosomes derived from bone marrow microenvironmental mesenchymal stromal cells (65). Barrera-Ramirez *et al* (65) have identified that mesenchymal stromal cell-derived exosomes in patients with AML demonstrate diminished levels of microRNA (miR)-339-3p, a direct RRBP1 suppressor, thereby derepressing RRBP1 transcription. This finding not only expands the oncogenic repertoire of RRBP1 beyond solid tumors, but also suggests a novel association between exosomal miRNA signaling and leukemic cell regulation. To the best of our knowledge, no studies have directly reported RRBP1 alterations or functional involvement in leukemias other than AML or in lymphomas, to date. Thus, its role in these hematological malignancies remains elusive and unreported, warranting further investigation.

In esophageal cancer, RRBP1 is highly expressed at both the mRNA and protein levels (10). Clinicopathological analysis has demonstrated that high RRBP1 expression is markedly and positively associated with the depth of tumor infiltration, LNM and advanced TNM stage (10). Patients with high RRBP1 expression exhibit a median survival of 43 months, in contrast to the 56 months observed in low-expression cohorts, thereby further validating its prognostic utility (10).

In bone metastasis, RRBP1 secreted by cancer cells exerts a paradoxical role in modulating osteoblast differentiation (66). Chen *et al* (66) have identified that RRBP1 is the only highly abundant shared protein associated with osteoblast differentiation in conditioned media of breast and prostate cancer cells. Knockdown of RRBP1 in cancer cells enhances osteoblast differentiation markers (ALP and bone γ-carboxyglutamate protein), matrix mineralization and bone morphogenetic protein (BMP) 2/SMAD1/5/9 activation in pre-osteoblasts (66). These findings indicated that bone metastatic cancer cells inhibit osteoblastic differentiation by secreting RRBP1 and the mechanism may involve the regulation of ERS levels and interference with the BMP/SMAD signaling pathway. These findings provide a potential theoretical basis for targeting RRBP1 in the treatment of bone metastasis-associated bone lesions in the future.

**Figure 5.**
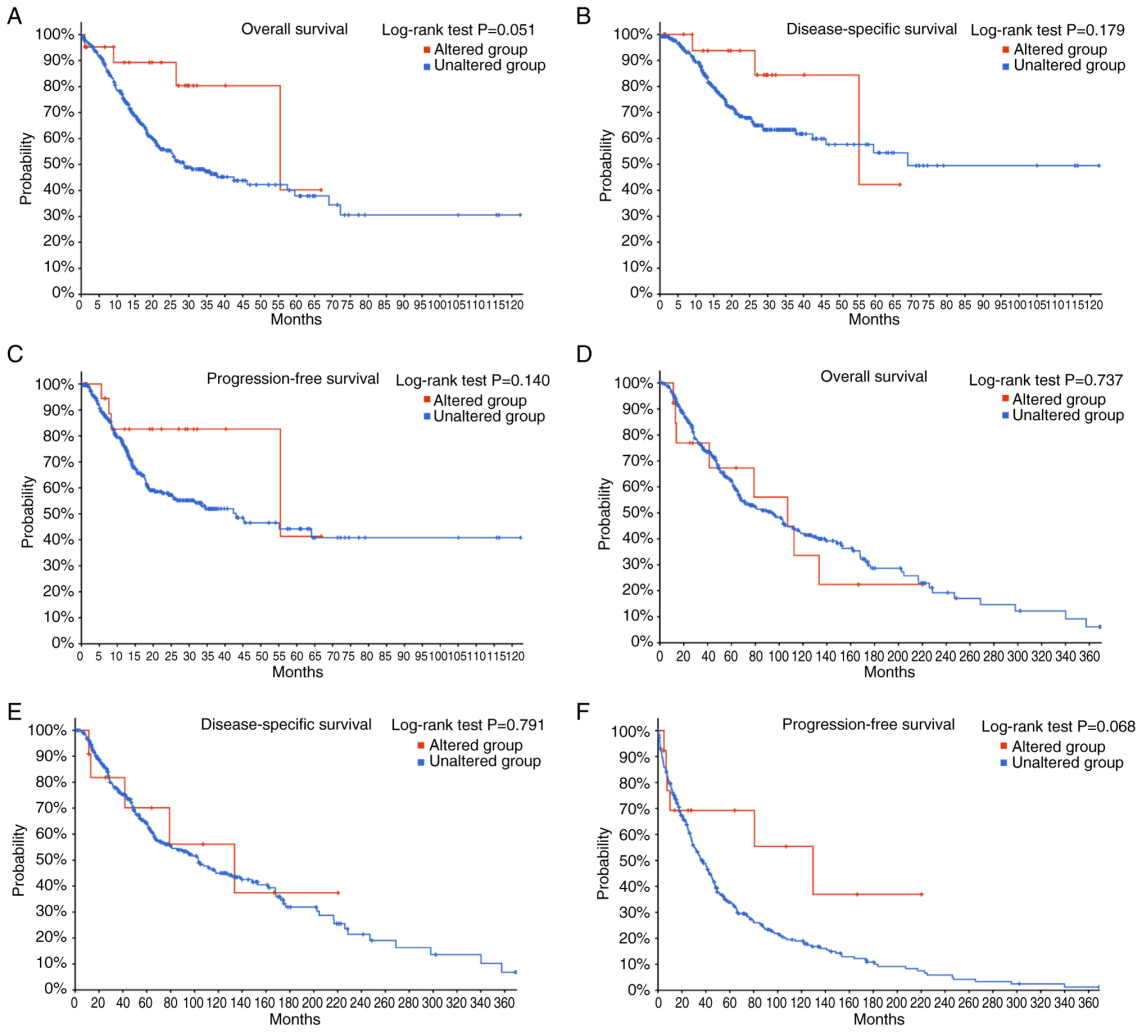
Survival analysis of *RRBP1* alterations in stomach adenocarcinoma and skin cutaneous melanoma (cBioPortal database). (A-C) Kaplan-Meier curves in patients with stomach adenocarcinoma with (red) or without (blue) *RRBP1* alterations. (A) Overall survival. (B) Disease-specific survival. (C) Progression-free survival. (D-F) Kaplan-Meier curves in patients with skin cutaneous melanoma. (D) Overall survival. (E) Disease-specific survival. (F) Progression-free survival. RRBP1, ribosome-binding protein 1.

Beyond the aforementioned cancer types, several tumor types have high *RRBP1* alteration frequencies but lack published evidence, to the best of our knowledge. Examples include stomach adenocarcinoma and skin cutaneous melanoma (Fig. 2). The present review used the cBioPortal database (TCGA; PanCancer Atlas; http://www.cbioportal.org/) to perform Kaplan-Meier survival analyses for these cancer types and compared the survival outcomes between patients with vs. without *RRBP1* mutation (Fig. 5). For stomach adenocarcinoma, the *RRBP1*-altered group, as compared with the unaltered group, demonstrated a trend toward improved OS, disease-specific survival and progression-free survival (Fig. 5A-C). Similarly, in skin cutaneous melanoma, patients with *RRBP1* alterations also showed a trend toward improved OS, disease-specific survival and progression-free survival (Fig. 5D-F). Although these comparisons did not reach statistical significance (log-rank P>0.05), the consistent direction of the curves suggested potential biological relevance. One possible explanation is that certain mutations in *RRBP1* may impair its function in protein transport and, therefore, slow tumor progression or they may occur in molecular subtypes that are intrinsically associated with a more favorable prognosis. However, due to the limited number of mutated cases and potential confounding clinical variables, these observations should be interpreted with caution and require further exploration in studies with larger sample sizes or functional studies.

## 4. Therapeutic targeting of RRBP1 in cancer

**Figure 6.**
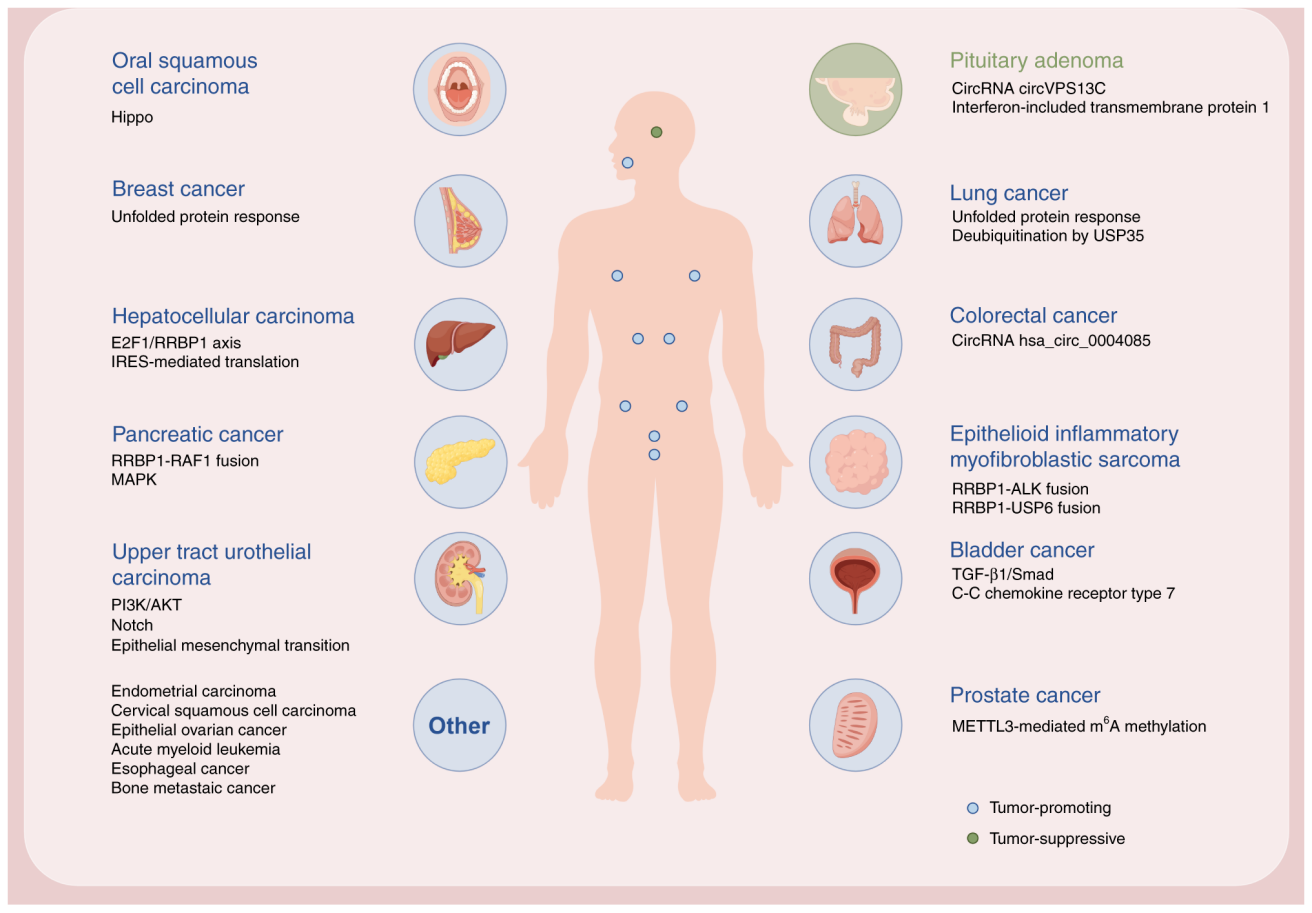
RRBP1 expression in various human organs and associated cancer types. Schematic diagram demonstrating RRBP1-associated cancer types and representative molecular mechanisms or keywords. Blue circles indicate tumor-promoting roles, whereas green circles indicate tumor-suppressive roles. ‘Others’ include cancer types without specific organ illustration (created in FigDraw). RRBP1, ribosome-binding protein 1, circRNA, circular RNA; E2F1, E2F transcription factor 1; IRES, internal ribosome entry site; USP35, ubiquitin-specific processing protease 35; m^6^A, N6-methyladenine; ALK, anaplastic lymphoma kinase; METTL3, methyltransferase-like 3.

The multifaceted roles of RRBP1 in tumorigenesis and progression underscore its key position as a metastable hub at organelle-membrane interfaces. RRBP1 serves complex and key roles in multiple important molecular mechanisms in various types of cancer (Fig. 6). Although its upregulation is closely associated with tumor progression, metastasis and chemotherapy resistance, its mechanistic basis reveals environment-dependent regulatory mechanisms; therefore, RRBP1 is a highly promising therapeutic target across cancer types.

### Targeting RRBP1-mediated stress adaptation

Stabilization of GRP78 inhibits ERS-associated apoptosis and decreases ROS accumulation, thus markedly enhancing cancer cell survival and chemoresistance (Fig. 3). In lung cancer, breast cancer and CRCs, RRBP1 stabilizes GRP78 mRNA, thereby enhancing UPR-mediated survival under ERS (4,36,46). Small molecules targeting the GRP78-PERK-eIF2α axis, such as PERK inhibitors (67), directly block the signaling pathway but have the drawback of pancreatic toxicity. Alternatively, by using a monoclonal antibody blocking RRBP1/GRP78 interaction, the regulation of UPR by RRBP1 can be decreased, thus reversing chemoresistance.

### Epigenetic modulation strategies

Enhancing self-stability through epigenetic modification or inhibition of degradation pathways such as the METTL3/m^6^A axis and USP35 deubiquitination promotes adaptive stress signals (Fig. 3). In lung cancer, the USP35 deubiquitination modification inhibits the proteasome-mediated degradation pathway (41), whereas in prostate cancer, METTL3 stabilizes RRBP1 through m^6^A modification (49). Therefore, inhibition of the METTL3/m^6^A axis, such as with small molecule peptides (68), decreases the stability of RRBP1 and blocks tumor progression. In addition, METTL3 inhibits programmed cell death-ligand 1 (PD-L1) expression through m^6^A modification and weakens the antitumor immune response (69). Inhibition of METTL3 remodels the tumor microenvironment and enhances the efficacy of programmed cell death protein-1/PD-L1 blockade therapy. Furthermore, small molecule inhibitors or gene editing systems targeting USP35 can also decrease RRBP1 protein stability (70). However, functional synergy among USP family members poses an important technical hurdle. Specific USP isoforms, such as USP7, USP14 and USP22, can promote therapeutic resistance by enhancing pro-survival protein stability, while other members may participate in distinct regulatory processes (70). This functional heterogeneity not only leads to a lack of specificity for targeted interventions but may also trigger unintended off-target effects. Therefore, in-depth analysis of the molecular basis of specific USP subtypes mediating drug resistance phenotypes will provide a key theoretical basis for the achievement of precision interventions in the future.

### Pathway inhibition approaches

Activation of the TGF-β/SMAD, PI3K/AKT, Notch and MAPK pathways, and inhibition of the Hippo pathway, drives tumor proliferation and anti-apoptotic ability (Fig. 4) (6,8,61,62). To address the multifaceted nature of the tumor, a layered targeting strategy can be designed. First, small molecule inhibitors or antibody drugs can be developed to specifically target RRBP1 and eliminate its inhibition of the Hippo pathway and synergistic activation effect of multiple pathways. Second, key downstream nodes can be targeted, e.g., with YAP/TAZ-TEAD complex blockers (71), dual PI3K/mTOR inhibitors (72), Ras/Raf/MAPK inhibitors (73) or neutralizing antibodies to decrease ligand-receptor binding (74,75). Third, epigenetic regulation can be integrated by co-delivery of RRBP1 small interfering RNA with DNA methyltransferase inhibitors via nanocarriers, thus reversing pro-oncogenic regulation. Notably, because the RRBP1 pathways of activation and inhibition differ among cancer types, precise guidance of treatment and localization of its subcellular mode of action are necessary, in conjunction with spatial transcriptomics technology to potentially achieve precise spatiotemporal targeting in the future.

### Fusion gene-targeted therapy

At the gene level, *RRBP1* is abnormally activated by gene fusions (*ALK, Raf1* and *USP6*) and subsequently regulates kinase function or deubiquitination; therefore, RRBP1 is a potential therapeutic target (Fig. 4) (56,60,61). For RRBP1-ALK/Raf1 fusion proteins arising in EIMS and pancreatic cancer, selective kinase inhibitors can impair aberrant kinase activity but must be optimized to overcome RRBP1-mediated drug resistance (73,76). For RRBP1-USP6 fusions with abnormal ubiquitination regulation, proteasome inhibitors or deubiquitinating enzyme inhibitors can be explored (70). In addition, the rapid development of gene editing technology in recent years is expected to overcome existing therapeutic bottlenecks and provide more effective treatment options through precise gene modification or modulation strategies (77–81). Notably, one EIMS case report stated that the *RRBP1-ALK* fusion^+^ case has notable histological features, predominantly collagenous mesenchyme with only localized areas of a mucus-like matrix (57). In contrast to the poor prognoses of previous *RRBP1-ALK* fusion cases, which resulted in rapid recurrence or death (56), this case demonstrated a favorable prognosis with no recurrence or metastasis during as many as 50 months of follow-up after crizotinib-targeted therapy (57). This prognostic difference may be closely associated with the characteristics of the collagen fiber-dominated microenvironment in the tumor stroma and the standardized use of ALK inhibitors after surgery. Therefore, the development of therapeutic strategies should be based on a combination of molecular features and tissue microenvironmental characteristics. In addition, the type of *ALK* fusion may be associated with the primary site of the tumor. *RRBP1-ALK* fusions are observed predominantly in abdominal EIMS, whereas rare fusion types such as echinoderm microtubule-associated protein-like 4-*ALK* and vinculin-*ALK* have been reported in central nervous system cases (82). This molecular heterogeneity highlights the need for molecular testing in the diagnosis and classification of EIMS, particularly for the differential diagnosis of cases involving rare sites. In summary, gene fusions reveal molecular heterogeneity and clinical diversity in different tumor types and the differences in pathogenic mechanisms, histological features and therapeutic responses highlight the key roles of precision medicine in diagnostic and therapeutic decision-making. Future studies should further clarify the interaction between the molecular characteristics of fusion genes and the tumor microenvironment and establish improved genotype-phenotype correlation models to potentially achieve truly personalized treatment.

### RNA-interaction disruption

RRBP1-RNA interactions have further revealed the complex roles of RRBP1 in the regulation of tumor adaptation (Fig. 4). In CRC, hsa_circ_0004085 binds RRBP1, stabilizes GRP78, enhances tumor cell adaptation to ERS and promotes drug resistance, thereby inhibiting apoptosis (46). For such RNA-interaction networks, the development of targeted antisense oligonucleotides (83), combined with specific sequence design, chemical modification optimization, efficient delivery systems and combined therapeutic strategies, can potentially overcome the stability challenges posed by the cyclic RNA structure, disrupt its binding to RRBP1 and restore apoptosis susceptibility. However, in NFPA, overexpression of RRBP1 partially reverses the inhibitory effect of circVPS13C on IFITM1, thereby inducing apoptosis (63). This duality challenges the traditional dichotomy of oncogenes and tumor suppressor genes, highlights the high context dependence of RRBP1 function and emphasizes the need to design layered intervention strategies based on microenvironmental characteristics.

The dual role of RRBP1 as a stress adaptor and fusion partner presents both opportunities and challenges. Targeting RRBP1-interacting pathways might enable precision therapy by relying on the vulnerability of the upper and lower pathways. However, both the tissue specificity of the RRBP1-ALK fusion and the tumor suppressor function of IFITM1 by maintaining it in NFPA caution against a one-size-fits-all solution (56–59,63). Future research can prioritize functional stratification of the RRBP1 network by using spatial multi-omics to determine how organelle communication is rewired in specific tumor subtypes.

Despite the identification of RRBP1 as a promising therapeutic candidate, to the best of our knowledge, no interventional clinical trials directly targeting RRBP1 have been registered in major trial repositories (ClinicalTrials.gov and World Health Organization International Clinical Trials Registry Platform) to date. Current therapeutic concepts remain largely preclinical and indirect, focusing instead on upstream or downstream regulatory nodes such as GRP78, METTL3, USP35 or oncogenic fusion partners (ALK, Raf1 and USP6) (67–70,73,76). These strategies underscore the translational potential of RRBP1 but also highlight that its development as a direct druggable target is still at an early investigational stage.

## 5. RRBP1 cancer-independent activities

Beyond oncology, the multifaceted roles of RRBP1 in diverse physiological and pathological settings highlight its key function as a structural and metabolic coordinator. In the musculoskeletal system, RRBP1 exhibits a regulatory role by driving collagen biosynthesis and extracellular matrix (ECM) remodeling in fibroblasts through miR-206-mediated post-transcriptional regulation (84). Furthermore, its dynamic expression during periodontal ligament stem cell osteogenesis underscores its potential to promote osteogenesis (85), possibly by promoting ER-dependent ECM secretion. Paradoxically, the observation that knockdown of RRBP1 in osteoblasts attenuates ALP and runt-related transcription factor 2 expression in an osteoporosis model suggests that bone formation is influenced by the environment (86). These findings have revealed that RRBP1 is a multi-effect regulator spanning the environment of ECM dynamics, osteogenic differentiation and bone formation. Therefore, RRBP1 function can be considered an environmentally responsive regulatory hub whose mode of action exhibits a dynamic balance between physiological tissue remodeling and pathological bone homeostatic imbalance.

Previous studies examining the relationship between RRBP1 and hepatic lipid metabolism have focused on the functional effects of RRBP1 expression regulation on ER-mitochondrial complex formation and lipid metabolic output (21,32–34). Although these functional gains or losses provide direct mechanistic insights into the regulatory ability of RRBP1, they may not fully reproduce the pathophysiological sequence observed *in vivo*, whereby systemic metabolic alterations precede disruption of lipid homeostasis. Notably, emerging investigations have begun to bridge this knowledge gap by interrogating RRBP1 dynamics under physiologically relevant metabolic states. For instance, a seminal study has systematically characterized fasting-induced RRBP1 expression and subcellular localization patterns in both lean and obese mouse models (87). Under fasting, RRBP1 is specifically upregulated around the hepatic portal vein and in the middle of the lobule and it drives specific remodeling of rough surface ER-coated mitochondria, thereby maintaining mitochondrial fatty acid oxidation function (87). By contrast, RRBP1 deficiency inhibits fasting-induced ER-mitochondrial interactions and leads to mitochondrial rounding, impaired β-oxidation and lipid accumulation (87). These results have revealed the molecular basis of RRBP1 in mediating metabolic adaptation through dynamic regulation of ER-mitochondrial spatial architecture. These findings have also clarified the role of RRBP1 as a key structural regulator that dynamically translates physiological stimuli or pathological injury into structural and functional remodeling of organelle networks. Future studies should prioritize the elucidation of the upstream signaling pathways regulating RRBP1 post-translational modifications and their spatiotemporal coordination with other membrane contact site regulators during metabolic adaptation.

## 6. Conclusions and perspectives

In cancer, RRBP1 drives malignancy by modulating molecular signaling pathways and associated organelles, such as by stabilizing the GRP78-PERK-eIF2α axis and inhibiting apoptosis, activating pro-survival pathways (TGF-β/SMAD and PI3K/AKT) and binding cyclic RNAs. In aggressive tumors, such as pancreatic cancer and EIMS, this protein oncogenic role is further amplified by fusion events (*RRBP1-ALK* and *RRBP1-Raf1*). Beyond cancer, RRBP1 serves key central roles in several physiological and pathological processes, such as the regulation of metabolic homeostasis, regulation of neuronal function, homeostasis of the cardiovascular system and remodeling of bone metabolism by regulating the interactions between cellular organelles.

However, clinical translation faces challenges of expression heterogeneity among cancer subtypes and the limitations of current models, which are unable to reproduce systemic effects due to a lack of cross-disease comparisons, since several previous studies have relied on *in vitro* systemic or single-tissue analyses (6,42,61,62). To bridge these gaps, future research efforts should prioritize multi-omics integration to decode the tissue-specific regulatory networks and immune microenvironmental interactions of RRBP1. Combining mechanistic understanding with advanced *in vivo* models (humanized organoids or multi-tissue CRISPR screens) could accelerate the development of organelle-targeted therapies and pan-disease biomarkers, thereby potentially transforming precision medicine from subcellular dynamics to clinical outcomes. Although the cancer-promoting mechanism of RRBP1 has been gradually revealed, its dynamic interaction network and targeted intervention strategies can be further explored in depth, particularly to overcome therapeutic resistance and microenvironmental remodeling. Therefore, there is an urgent need for translational research in this area.

## Supplementary Material

Supporting Data

## Data Availability

Not applicable.
