# Supplementary material for: Ribosome-binding protein 1: A multidimensional regulator of cancer progression and a novel target for precision therapy (Review)
Source: Oncol Lett. 2025 Oct 23;31(1):5. doi: 10.3892/ol.2025.15358 (PMC12587471; doi:10.3892/ol.2025.15358)

Figure S1. Domain organization and predicted 3D structure of the RRBP1-ALK fusion protein. (A) Schematic of RRBP1-ALK domain architecture. The RRBP1 portion contains a transmembrane region, ribosome receptor, RPT1 and coiled-coil/low-complexity segments; the ALK portion retained in the fusion comprises the TyrKc only. The dashed line denotes the fusion breakpoint. The sequence was retrieved from Ensembl (https://www.ensembl.org/, release 114) and its domain annotations were obtained from SMART(http://smart.embl.de/). (B) Predicted 3D model generated with AlphaFold server (https://alphafoldserver.com/). The blue-to-yellow gradient indicates the predicted local distance difference test confidence score (blue, high; yellow/orange, low). The model highlights extended α-helical/coiled-coil elements from RRBP1 adjacent to the globular ALK kinase domain, consistent with a structural basis for dimerization-driven kinase activation. RRBP1, ribosome-binding protein 1; TyrKc, tyrosine kinase catalytic domain; ALK, anaplastic lymphoma kinase; Rib_recp_KP_reg, ribosome receptor lysine/proline rich region; RPT, internal repeats; MAM, meprin-A5-PTPμ domain; LDLa, low-density lipoprotein receptor domain class A.

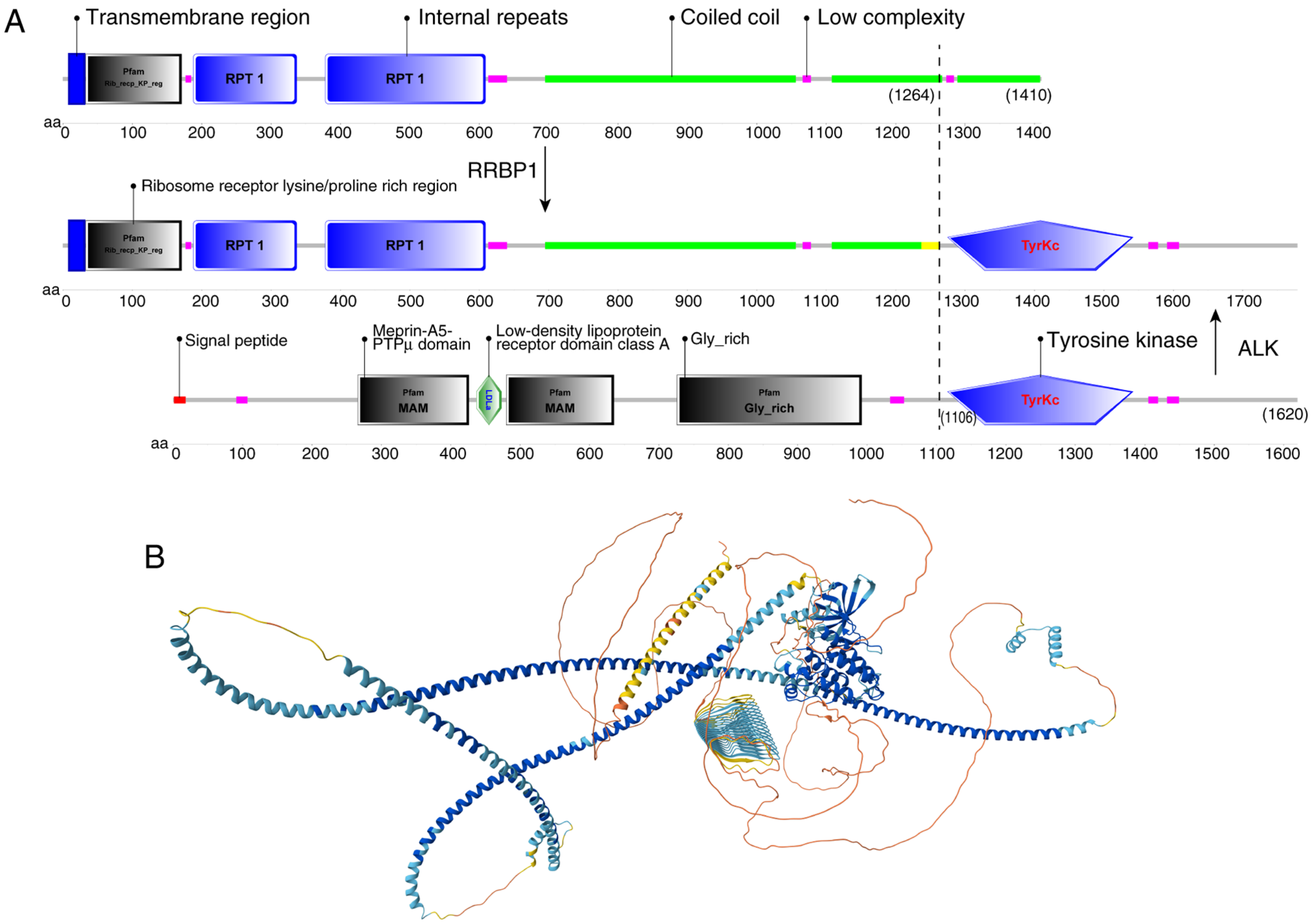

Supplement: Supporting Data [file Supplementary_Data.pdf]
